# Lectures to Practitioners

**Published:** 1888-09

**Authors:** 


*Lectures to Practitioners.* Tabes Mesenterica, by W. T. GAIRDNER, M.D.; and The Pathology of Phthisis Pulmonalis, by JOSEPH COATS, M.D. Pp. 286. London: LONGMANS & CO. 1888.

Glasgow practitioners are fortunate in having such men as Dr. Gairdner and Dr. Coats to lecture to them on subjects with which they are specially familiar. The Lectures are evidently the outcome of much experience and research on the part of both authors. As an advanced philosophic and clinical study of the whole subject of consumption, the work is of great value, and deserves to take a permanent place in the literature of the subject.

